# Synthesis and characterization of visible-light-driven novel CuTa_2_O_6_ as a promising practical photocatalyst

**DOI:** 10.3389/fchem.2023.1197961

**Published:** 2023-06-22

**Authors:** Krishnaprasanth Alageshwaramoorthy, Pandian Mannu, Seetha Mahalingam, Ta Thi Thuy Nga, Han-Wei Chang, Yoshitake Masuda, Chung-Li Dong

**Affiliations:** ^1^ Department of Physics, Kongunadu Arts and Science College, Coimbatore, India; ^2^ Research Center for X-ray Science and Department of Physics, Tamkang University, Tamsui, Taiwan; ^3^ Department of Chemical Engineering, National United University, Miaoli, Taiwan; ^4^ Pesticide Analysis Center, National United University, Miaoli, Taiwan; ^5^ National Institute of Advanced Industrial Science and Technology (AIST), Nagoya, Japan

**Keywords:** phase transition, photocatalytic activity, dye degradation, X-ray absorption spectroscopy (XAS), visible light irradiation

## Abstract

In this work, the novel CuTa_2_O_6_ phase was successfully synthesized by the hydrothermal and followed by the calcination process. The X-ray diffraction pattern confirms the formation of different phases. At a low temperature, CuTa_2_O_6_ exhibits the orthorhombic phase, whereas, at a higher temperature, it underwent a phase transition to a cubic crystal structure. X-ray photoelectron spectroscopic results suggest the presence of all the elements (Cu, Ta, and O). The optical studies were carried out using a UV-Vis DRS spectrophotometer. FESEM images confirm the spherical-shaped particles for the sample annealed at a high temperature. The local atomic and electronic structures around Cu and the contribution of the Cu oxidation state in the CuTa_2_O_6_ system were determined by X-ray absorption spectroscopy. To investigate the effective usage of CuTa_2_O_6_ in treating wastewater, its photocatalytic activity was investigated by evaluating its use in the photodegradation of MO dye under visible light irradiation. Moreover, the prepared CuTa_2_O_6_ photocatalyst exhibits significant photocatalytic activity in the degradation of MO dye and shows excellent stability; it is therefore a promising material for potential use in a practical photocatalyst. The CuTa_2_O_6_ photocatalyst suggests an alternative avenue of research into effective photo-catalysts for solar hydrogen water splitting.

## 1 Introduction

Rapid population and industrial growth have made the environment a serious problem globally. Clean water is important for global and economic development, and the agricultural, industrial, and domestic sectors require huge quantities of unpolluted water. Over the last few decades, polluted water has caused major problems, especially for aquatic organisms, animal ecosystems, and humans (Gheytanzadeh et al., 2022). Notably, the dyes from textile, leather, fabric, paper, cosmetics, pulp, food, dye synthesis, plastics, and pharmaceuticals generate wastewater that emerges as threatening pollutants to the environment (Oyewo et al., 2022). Just a few layers of dye on the surface of the water blocks sunlight and disturb the photosynthesis of aquatic species below (Abdelrahman et al., 2019). Primary techniques such as coagulation, filtration, reverse osmosis, and flocculation can be used to remove dye from wastewater. Instead of degrading the pollutants; these techniques simply convert the pollutants from one form to another, causing secondary pollution (Ajmal et al., 2014). Therefore, many methods have been employed for wastewater treatment such as chlorination, flocculation, ozonation, adsorption, photocatalysis, chemical oxidation, and biodegradation (Hodges et al., 2018; Yang et al., 2020; Sahoo et al., 2022). Among, advanced oxidation processes (AOPs) such as H_2_O_2_/UV processes, and photodegradation are commonly used for clean energy and biodegradable catalysts for the removal of pollutants. Additionally, the photocatalyst includes the whole mineralization of organic pollutants into carbon dioxide and water without any harmful by-products is being as the most promising technique (Yang et al., 2020) (Sahoo et al., 2022).

Heterogeneous photocatalysis using semiconductors is a broad field that involves dye photodegradation from wastewater (Majhi et al., 2021; Louis et al., 2022). TiO_2_ is a well-known photocatalyst whose crystalline structure can be modified to increase its photocatalytic activity in water treatment. However, the large bandgap of anatase TiO_2_ (3.2 eV) limits its efficiency under solar light: it uses only 4% of the total solar spectrum (Rose et al., 2022). Therefore, a more efficient, chemically stable photocatalyst that is active in the visible light region must be developed. Various semiconductors like ZnO, Bi_2_O_3_, and SnO_2_, as well as also metal-chalcogenides like Cds, CdSe, CdTe, and Pbs have been investigated for this purpose (Djurišić et al., 2014; Ramchiary, 2020; Li et al., 2023). Several strategies have been used to improve their photocatalytic activity, doping or coupling of low bandgap semiconductors with suitable materials can effectively improve photocatalytic activity under visible light (Zhang et al., 2006; Zhou et al., 2009; Xu et al., 2012a). It is well known that copper-based metal oxides are p-type semiconductors, commonly used in solar energy applications owing to their narrow band gap of ∼ 1.2 eV. Compared to other regularly used p-type materials like NiO, it exhibits higher charge carrier motilities (100 cm^2^v^−1^s^-1^) while NiO has (0.53 cm^2^v^−1^s^-1^). They are more stable in aqueous media than Group III-V or II-VI semiconductors (Sullivan et al., 2016). The narrow band gap and good stability of copper make it suitable for photocatalytic applications (Sibhatu et al., 2022).

On the other hand, great interest has been shown in the photocatalytic activity of Tantalum-based materials that include Ta_2_O_5_, TaON, and Ta_3_N_5_ (Zhang et al., 2014; Suzuki et al., 2017; Gomes et al., 2018). Apart from these materials, transition metal tantalates and antimonates (general formula M′M″ _2_O_6_, where: M′ = Cu/Zn, and M′′ = Ta/Sb) emerging in various research due to their excellent magnetic, electrical, optical, thermoluminescence and more importantly the catalytic properties (Nguyen et al., 1996; Kato et al., 2002; Dutta et al., 2013; Noto et al., 2013). These composites represent an important group of semiconductors, characterized by high thermal stability (above 1,000 °C) and ease of modification of their physicochemical properties. Therefore, among these materials, CuTa_2_O_6_ has been a promising candidate in fundamental research and numerous applications (Nguyen et al., 1996; Kato et al., 2002; Dutta et al., 2013; Noto et al., 2013). Available literature suggests that the CuTa_2_O_6_ compound is formed in the binary system of CuO-Ta_2_O_5_ oxides (Golubev et al., 2017). Moreover, CuTa_2_O_6_ also occurs in two polymorphic varieties which include monoclinic and tetragonal (Krabbes and Langbein, 1996). Some authors reported that there is an orthorhombic variety of this CuTa_2_O_6_ compound as well (Vincent et al., 1978). Even though many studies have been carried out on CuTa_2_O_6_ that include high dielectrics, exceptional photoelectric performance, and chemical stability, there are only limited investigations done on the photocatalytic properties of CuTa_2_O_6_.

This work demonstrates the fundamental parameters involved in the photocatalytic activity of copper tantalite, which can be used as a water-splitting material due to its promising properties. To the best of the authors’ knowledge, photodegradation of MO dye under visible light irradiation on the CuTa_2_O_6_ phase has not been previously investigated. Thus, a novel CuTa_2_O_6_-based photocatalyst is prepared by a hydrothermal and calcination process, and its photocatalytic performance under visible light irradiation is studied.

## 2 Materials and methods

All of the chemical reagents including copper nitrate trihydrate (Cu (NO_3_).3H_2_O) and tantalum chloride (TaCl_5_), were purchased from Sigma Aldrich (United States) and used without further purification. De-ionized water was used as a solvent throughout the experiment and ethanol was used to wash the prepared photocatalyst. Methyl orange (MO) dye was purchased from Merck and used as a model pollutant.

In a typical synthesis procedure, an equal amount (0.1M) of tantalum chloride and copper nitrate trihydrate was dissolved in 30 mL of de-ionized water, which was then stirred continuously at room temperature to yield a homogeneous solution. Then, this solution was transferred to a 50 mL Teflon-lined beaker and kept in a stainless-steel autoclave at 180°C for 8 h. After attaining room temperature, the obtained product was washed several times with de-ionized water and ethanol followed by drying at 80°C for an hour. The dried powder was collected and annealed at 500°C, 700°C, and 900°C.

### 2.1 Characterization

The crystal structure and the phase purity of the prepared samples were confirmed by X-ray diffraction (Shimadzu 6,000) with Cu-Kα radiation, λ = 1.5406 Å. The surface morphology was analyzed using a field emission scanning electron microscope (FESEM model—CARLZEISS SIGMA HV). The chemical composition analysis was studied by Thermo Scientific K-Alpha X-ray Photoelectron Spectroscopy (using ESCA-3400 XPS from Kratos Analytical, Shimazu). The optical band gap was estimated from UV-Vis diffused reflectance spectra using the JASCO V-670 double-beam spectrophotometer model. Synchrotron X-ray absorption spectra (Cu K-edge), including X-ray absorption near edge structure (XANES) and extended X-ray absorption fine structure (EXAFS) spectra, were obtained at BL17C of the National Synchrotron Radiation Research Center (NSRRC), Taiwan. The specific surface area was calculated from N_2_ adsorption-desorption isotherms measured using the ANTON PAAR NOVATOUCH LX2 (Graz, Austria), using the Brunauer–Emmett–Teller (BET) equation.

### 2.2 Photocatalytic experiment

The photodegradation of MO dye was examined in the presence of the prepared CuTa_2_O_6_ photocatalyst. In a typical experiment, 50 mg of the photocatalyst was added to 100 mL of aqueous MO with an initial concentration of 10 mg/L. Before irradiation, the mixture was continuously stirred at room temperature in the dark for 30 min to achieve an adsorption/desorption equilibrium. Then, the mixed solution was irradiated with visible light using a 75 W high-pressure mercury vapor lamp. During the irradiation, about 2 mL of the suspension was taken at regular intervals (10 min) and analyzed using the UV-Vis spectrophotometer to determine the concentration of MO dye; the characteristic absorption of MO was about 464 nm (Mageshwari et al., 2013).

## 3 Results and discussion

### 3.1 Structural analysis

**FIGURE 1:**
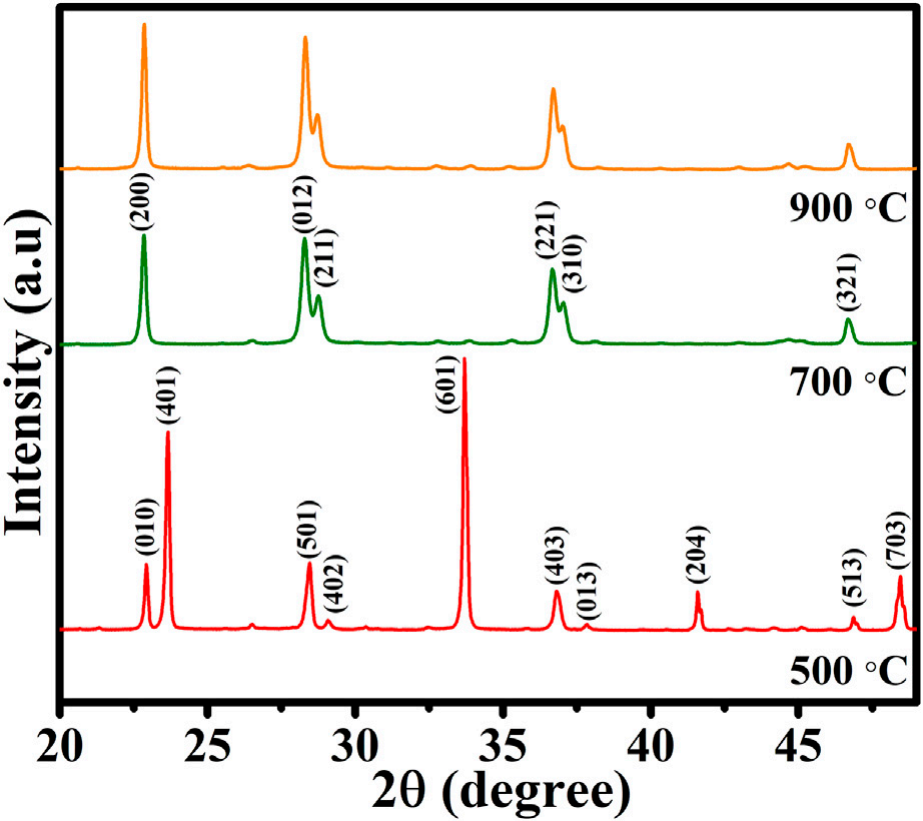
XRD patterns of CuTa_2_O_6_ annealed at different temperatures (500°C, 700°C, and 900°C).

Figure 1 shows XRD patterns of the prepared CuTa_2_O_6_ at different annealing temperatures. The diffraction peak reveals the polycrystalline nature of the prepared CuTa_2_O_6_ phase. The sample annealed at 500°C exhibits the orthorhombic phase of CuTa_2_O_6_ and agrees with JCPDS card no: 87–0,357. Then, increasing the annealing temperature to 700°C led to the formation of a different phase. The observed diffraction peaks are associated with the cubic phase of CuTa_2_O_6_, which agrees with JCPDS card 70–0,611. Further increase in the annealing temperature to 900°C did not modify the phase formation. Therefore, increasing the annealing temperature significantly affects the phase formation of CuTa_2_O_6_. The intensity of the diffraction peaks is higher, and the full width at half maximum (FWHM) is also slightly increased with an increase in the annealing temperature. The average crystallite size was calculated using the Debye-Scherer formula D = $\frac{K\lambda }{\beta \cos\theta }$ where K is the shape factor of the particles that are commonly called the Scherer constant (k = 0.89); λ is the wavelength of the X-ray radiation (*λ* = 0.1542 nm Cu-Kα radiation); θ is the Bragg angle, and β is the FWHM of the peak.

The difference in the XRD peak width is small between 700°C and 900°C. However, the actual variation is shown in Supplementary Figure S1 (supporting information), which evidences the increased value for full width at half maximum (FWHM). The intensity of the diffraction peaks and the FWHM) also increased as the temperature increased. The higher the FWHM, the smaller the crystallite according to the Debye-Scherer formula. Therefore, the calculated average crystallite size is found to be 32.28 nm, 29.79 nm, and 28.13 nm for the samples that were annealed at 500°C, 700°C, and 900°C respectively. An increase in the annealing temperature reduces the crystallite size, this could be due to the higher annealing temperature which might produce a relatively large temperature gradient within the submicrosheres, resulting in the formation of solid/liquid biphase core-shell structures through the non-equilibrium heating process (Tao et al., 2013). As heterogeneous nucleation only needs a low activation energy, the co-existence of heterogeneous phases can be helpful for the crystalline nucleation of CuTa_2_O_6_. Also, it is believed that the more generated nuclei, the smaller the crystallite size.

### 3.2 Morphological studies

**FIGURE 2:**
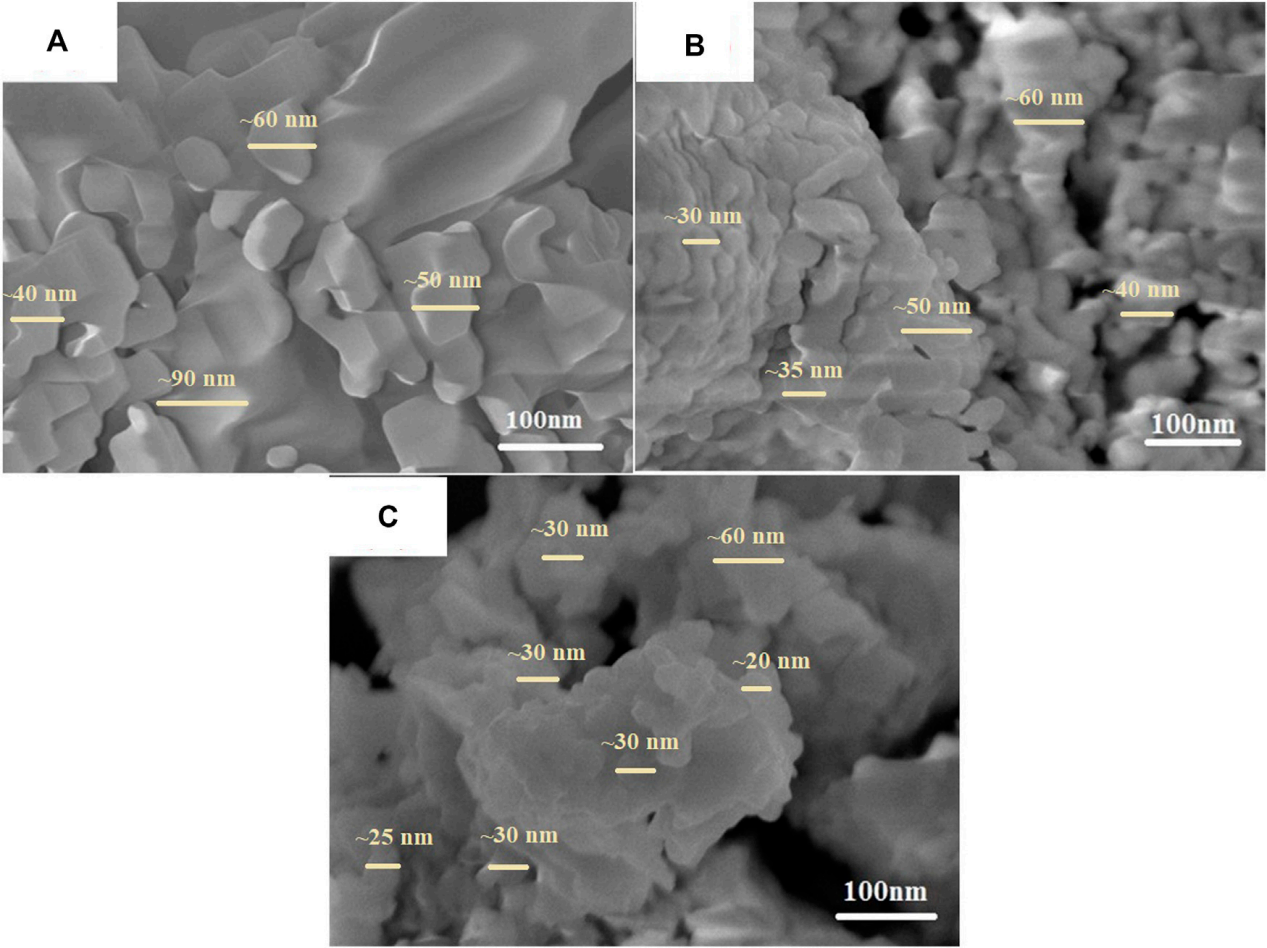
FESEM images of CuTa_2_O_6_ samples annealed at **(A)** 500°C, **(B)** 700°C, and **(C)** 900°C.

Figure 2A displays the nearly spherical shaped structure and uniform in size for the sample annealed at 500°C. It seems an increase in the annealing temperature to 700°C lead to agglomeration of these particles, as shown in Figure 2B. Further increase in the annealing temperature (900°C) produces more agglomerated spherical-shaped particles with irregular distribution throughout the surface. As a result of the higher temperature, irregular particle shapes are fused via adhesion.

Similar to the estimated average crystallite size from the XRD results, the particle is also assumed to be smaller in the sample annealed at 900°C as the particles may be composed of an agglomeration of many crystallites (as marked in Figure 2), which results in the formation of irregular and deformed particle shapes at higher annealing temperatures. Consequently, the smaller the crystallite size, the smaller the particle size. From Figures 2A–C, it seems the grains are dissimilar in different regions. Therefore, the particle size is likely to be different among the samples annealed at 500°C, 700°C, and 900°C as pointed out in Figure 2. For more evidence, the estimated average grain size is about 70 nm for a 500°C annealed sample. Then, it seems the average grain size gets reduced to ∼40 nm at 900°C and is smaller than the sample annealed at 700°C (∼50 nm). This implies that these particles/grains observed in the SEM image are composed of many crystallites/grains. Further, it is believed that the SEM image of the sample annealed at 900°C has composed of small size particles/grains than the other annealed samples.

### 3.3 X-ray photoelectron spectroscopy analysis

**FIGURE 3:**
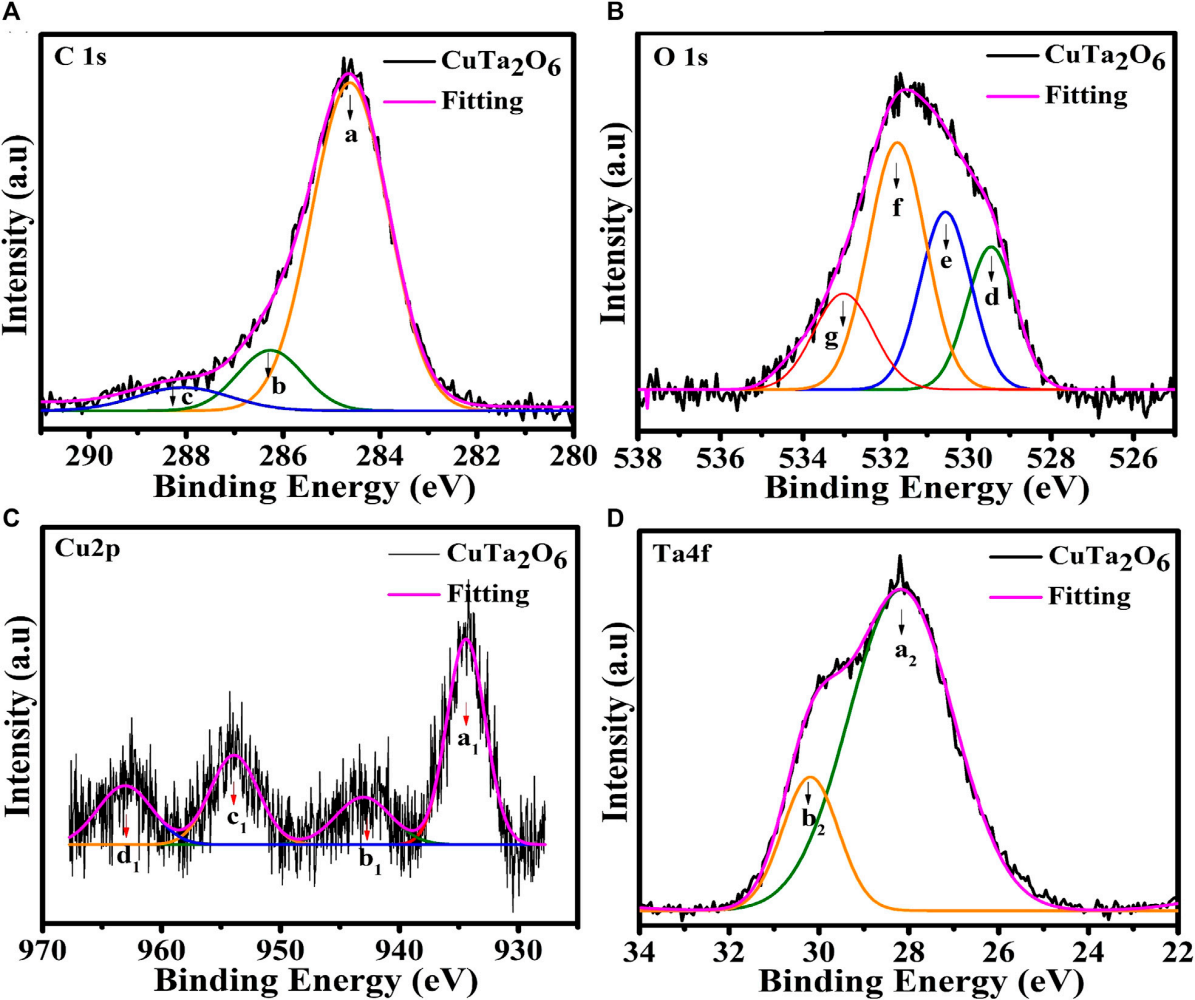
XPS analysis of CuTa_2_O_6_ annealed at 900°C **(A)** C 1s, **(B)** O 1s, **(C)** Cu2p, and **(D)** Ta4f.

The elemental composition and valence state of the prepared samples were determined by the XPS technique. Figure 3 shows the recorded XPS data of the sample that was annealed at 900°C. The peak features a, b_,_ and c are deconvoluted from the recorded C 1s spectra as shown in Figure 3A. The characteristic feature at 284.6 eV is sp2-hybridized carbon (C-C) that was adsorbed on the surface of the prepared sample (Khalid et al., 2013). Additionally, peaks at 286.2 eV, and 288.1 eV are related to the hydroxyl carbon (C-O), and carboxyl carbon (O=C-O), respectively (Liang et al., 2017). The measured O 1s spectra were deconvoluted into three peaks (Features d, e_,_ and f), as shown in Figure 3B. The presence of various oxygen ions in CuTa_2_O_6_ with potentially different coordination environments (more Cu-coordinated or more Ta-coordinated), accounts for the deconvolution of the O 1s signals into multiple components. A peak at 529.6 eV (feature d) could be related to the CuO lattice oxygen peak as the binding energy of the lattice oxygen for the bulk samples was frequently found to be slightly higher than that for the nanostructures (Svintsitskiy et al., 2013). Furthermore, the peaks at 530.6 eV and 531.7 eV (features e and f) are associated with Ta−O and Ta=O, respectively. This main peak (feature f) could be attributed to the bulk oxygen ions affected by charge imbalance due to the presence of oxygen vacancy. An additional shoulder peak at 533.0 eV (feature g) is attributed to an oxygen species that is coupled to hydroxyl or hydrated molecules. Consequently, the prepared CuTa_2_O_6_ phases may contain O-H groups (Chennakesavulu and Ramajaneya Reddy, 2015). The high-resolution pattern of Cu 2p as displayed in Figure 3C exhibits the characteristic peaks at 934.2 eV (a_1_ feature) and 954.1 eV (c_1_ feature) that correspond to Cu 2p_3/2_ and Cu 2p_1/2_, respectively, confirming the presence of Cu^2+^. The two other peaks at 943.1 eV (b_1_ feature) and 962.3 eV (d_1_ feature) are identified as shake-up satellite peaks of Cu 2p_3/2_ and Cu 2p_1/2_ respectively. The shake-up peaks may have arisen from an unfilled Cu 3d^9^ shell, suggesting the presence of Cu_2_O and Cu^2+^ in the prepared sample (Chennakesavulu and Ramajaneya Reddy, 2015) (Muthu Gnana Theresa Nathana et al., 2018). Figure 3D shows two peaks of Ta 4f_7/2_ (a_2_ feature) and Ta 4f_5/2_ (b_2_ feature) at 28.2 eV and 29.5 eV indicate the presence of tantalum in the Ta^5+^ oxidation state (Wang et al., 2012).

### 3.4 X-ray absorption spectroscopy (XAS) analysis

**FIGURE 4:**
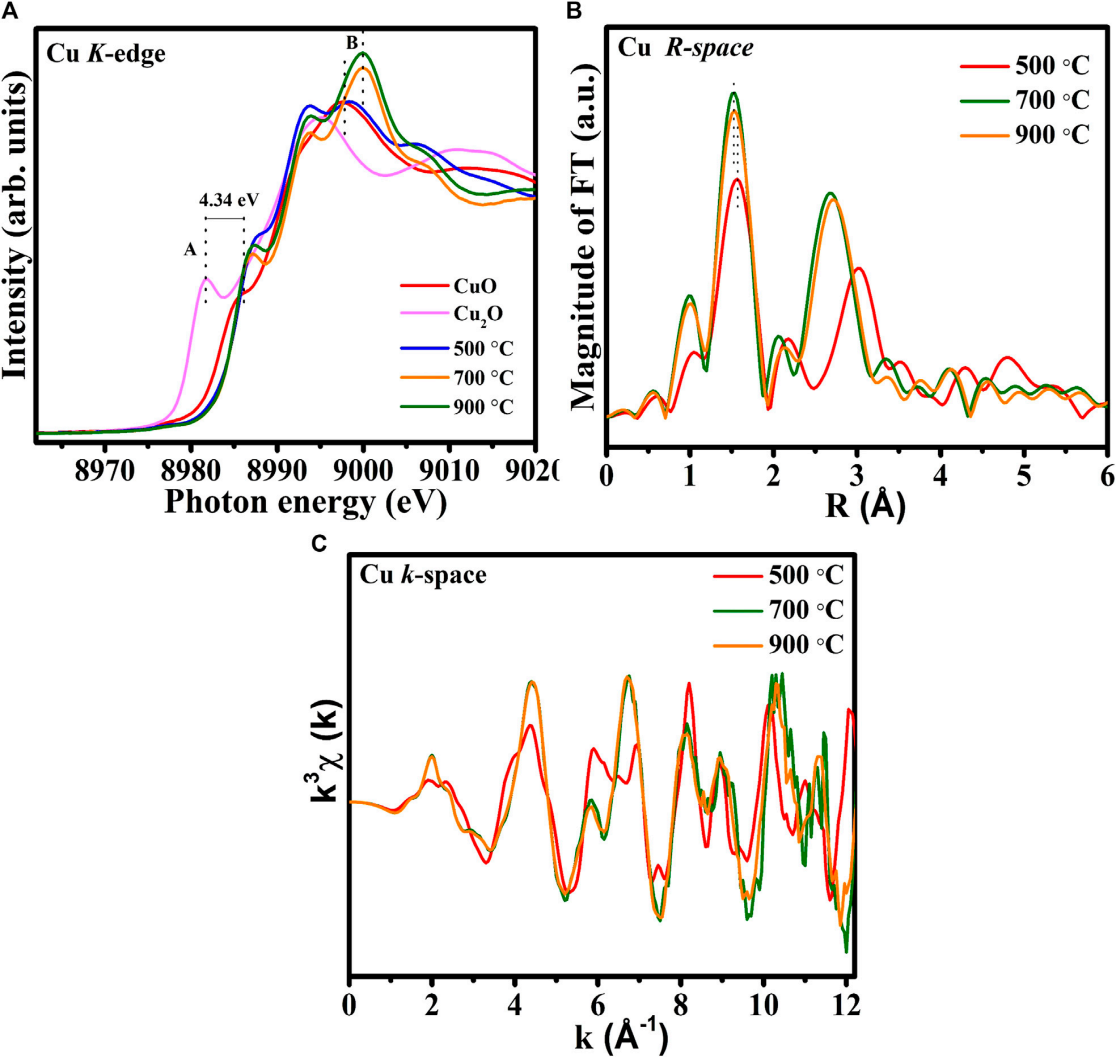
**(A)** Normalized Cu K-edge spectra of CuTa_2_O_6_ annealed at 500°C, 700°C, and 900°C, **(B)** corresponding Fourier-transformed EXAFS spectra, **(C)** EXAFS oscillations with k^3^-weighted χ**(K)** functions.

Figure 4A compares the Cu K-edge XANES spectra of the 500°C-, 700°C-, and 900°C-annealed CuTa_2_O_6_ phase with those of CuO (+1 oxidation state) and Cu_2_O (+2 oxidation state) references. The rising-edge peak at ∼8,980 eV for Cu_2_O reveals the oxidation state of Cu^1+^ (Kau et al., 1987). The absence of a pre-edge peak appears in the Cu K-edge XANES spectra is consistent with previous reports (Bera et al., 2002) (Khemthong et al., 2013). The main absorption peak around ∼9,000 eV reveals the 1s-4p transition, which merges into the continuum states (Khemthong et al., 2013). A shoulder peak (feature A) in the energy range 8,985–8,990 eV is attributed to the 1s → 4p_z_ transition (shakedown). The energy shift of this feature (feature A) from Cu_2_O to CuO is about 4.34 eV suggesting the valence state change from Cu^1+^ to Cu^2+^. The Cu K-edge spectra of CuTa_2_O_6_ have two regions that include a shoulder peak and a main absorption peak (feature A and feature B). It seems the peak feature (feature A) of all the annealed CuTa_2_O_6_ is close to that of CuO, revealing the oxidation state of Cu^2+^ (Kau et al., 1987) (Lim et al., 2019). Then, the main absorption peak (feature B) of annealed at 500°C is also similar to those CuO reference samples again confirming the oxidation state of Cu^2+^. However, the main absorption peak (feature B) of annealed 900°C shows a higher intensity compared to the annealed 700 °C suggesting the existence of more oxidation state of Cu^2+^. Also, this main absorption peak of located at a higher energy than the sample that was annealed at 500°C (feature B) so it is believed to have a more oxidation state.

To determine variations in the local atomic environment around Cu in the CuTa_2_O_6_ catalysts, the Fourier-transformed k^3^-weighted Cu K-edge spectra were obtained, as shown in Figure 4B. From the 500°C-annealed CuTa_2_O_6_, the major peaks at 1.56 Å and 3.01 Å correspond to Cu-O and Cu-Cu bonds, respectively (Nasir et al., 2017). The slight contraction in Cu-O bond length and the <comment > shortening in Cu-Cu/Ta bond at </comment>900°C indicates the presence of more oxygen vacancies and cationic vacancies. Commonly, the contracted bond lengths are associated with an increase in the oxidation state. Therefore, the slight contraction in the Cu-O bond at 900°C compared to the 500°C, is also accompanied by the increased oxidation state of Cu. This contraction in the Cu-O bond also suggests the higher coordination number in Cu-O first shell. It is believed that this contraction in Cu-O bond length could be due to the strong interfacial interaction among the Cu-O-Ta materials. It is assumed that the oxidation state of Ta is also to be different in these samples. Particularly, an increase in annealing temperature might lead to an alteration in the valence state of the absorbing atoms in the sample annealed at 900°C. This considerable variation in the coordination of Cu-O and Cu-Cu/Ta suggests the phase transformation of CuTa_2_O_6_, as evident from the structural analysis. The FT-EXAFS oscillations of the CuTa_2_O_6_ catalysts exhibit different profiles specifically for sample annealed at 500°C, suggesting dissimilar coordination environments of the Cu-O sites as presented in CuTa_2_O_6_ in Figure 4C.

### 3.5 Optical studies

**FIGURE 5:**
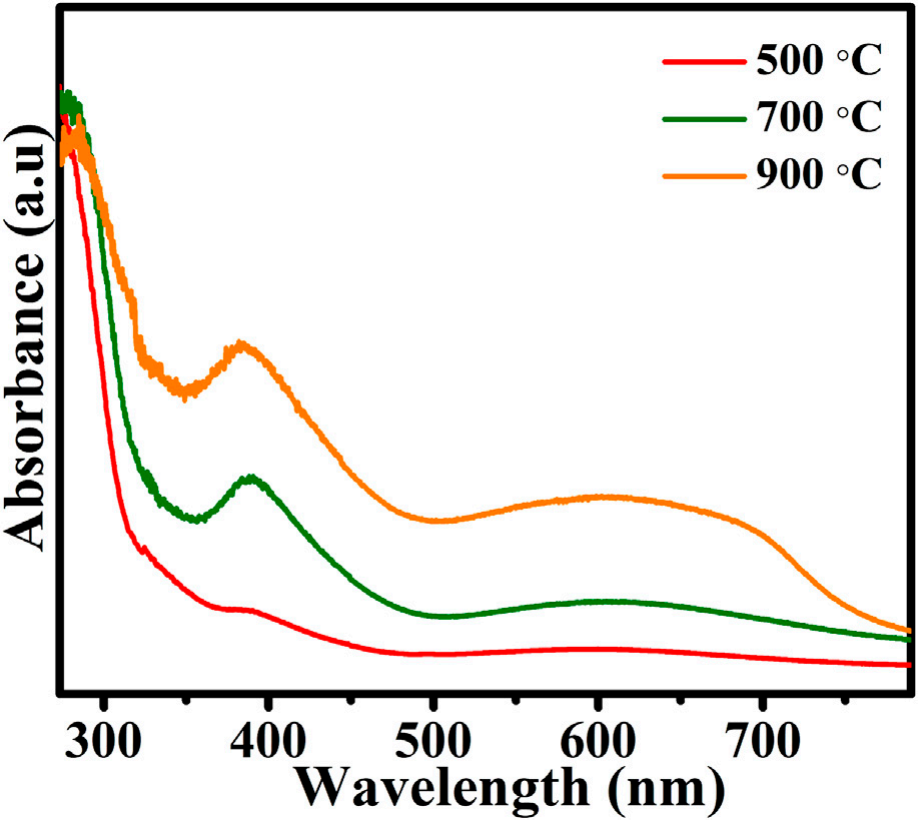
UV-Vis DRS analysis of CuTa_2_O_6_ annealed at different temperatures.

The UV-Vis DRS of the prepared CuTa_2_O_6_ phase was analyzed and is shown in Figure 5. All the prepared samples present a broad absorption range in the visible light region. The redshift that was observed with an increase in annealing temperature suggests that annealing increases sensitivity to visible light. The bandgap energy (E_g_) of each prepared catalyst was estimated using the relation E_g_ = 1,240/λ_g_ (eV), where λ_g_ is the absorption edge, which was obtained from the intercept between the tangent of the absorption curve and the abscissa. The estimated band gap energy of the samples that were annealed at 500°C, 700°C, and 900°C corresponds to 3.85 eV, 3.66 eV, and 3.26 eV, respectively. A decrease in the bandgap energy is observed for the sample annealed at a higher temperature (900°C). This could be attributed to the transition of the band from Cu 3d^+^ O 2p orbital to Ta 5d orbital as Cu 3d^10^ state may contribute to the valence band of the semiconductor, as similar behavior also reported on the materials such as metal oxides and sulfide photocatalysts (Xu et al., 2012b).

### 3.6 Photocatalytic activity

**FIGURE 6:**
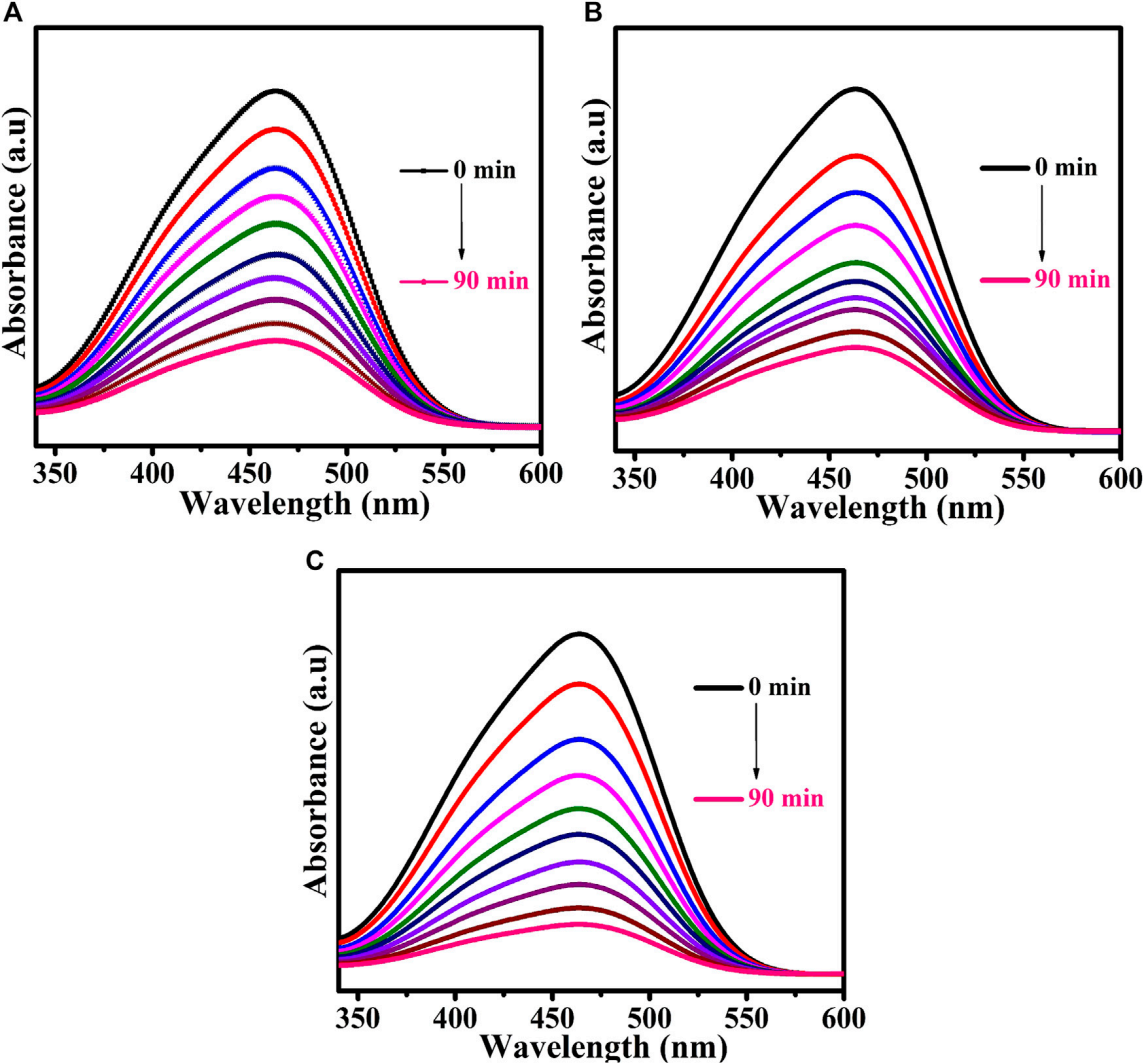
Time-dependent UV-Vis absorption spectra of MO dye solution without the addition of H_2_O_2_ for samples annealed at **(A)** 500°C, **(B)** 700°C, and **(C)** 900°**(C)**.

**FIGURE 7:**
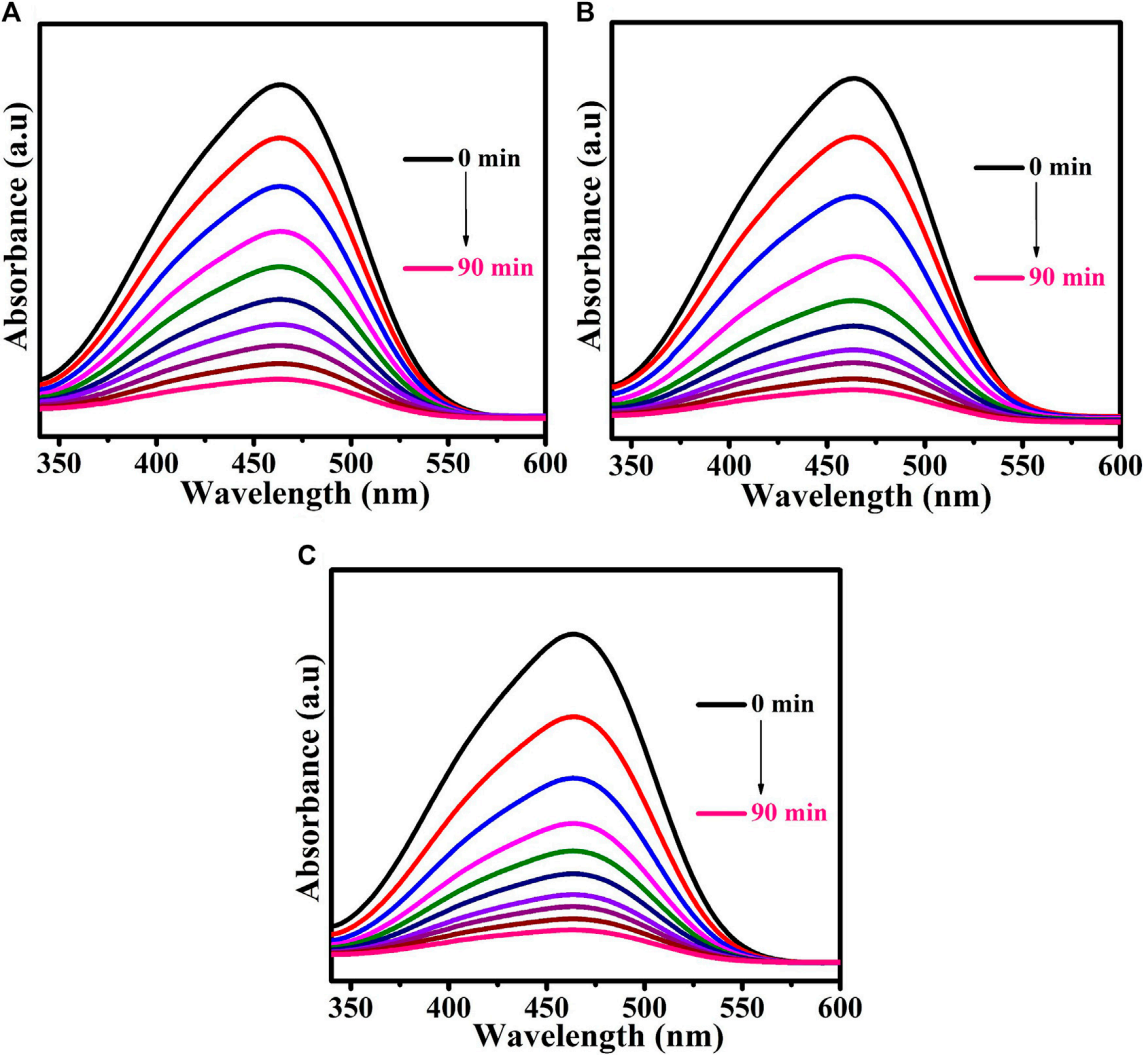
Time-dependent UV-Vis absorption spectra of MO dye solution for samples annealed at **(A)** 500°C, **(B)** 700°C, and **(C)** 900°C following the addition of H_2_O_2_.

Methyl orange dye was used as a model pollutant. The UV-Vis DRS analysis demonstrated that the prepared CuTa_2_O_6_ phase was active in the visible region. The photocatalytic process was carried out under visible light irradiation. Figure 6 shows the time-dependent UV-Vis absorption spectra of MO dye solution without and with the addition of H_2_O_2_ for the samples annealed at different temperatures (a) 500°C, (b) 700°C, and (c) 900°C. The UV-Vis DRS spectra of the MO dye solution show a characteristic peak at 464 nm. From Figure 6; Figure 7, the intensity of this characteristic peak gets decreases as the duration of exposure to light increases. At 90 min of exposure, the intensity of the peak was significantly reduced in comparison with 0 min, and the aqueous solution was colorless, indicating the decomposition of the dye molecules. The continuous disappearance of the absorption band suggests that the functional group of the MO dye is broken down (Xu et al., 2012b).

**FIGURE 8:**
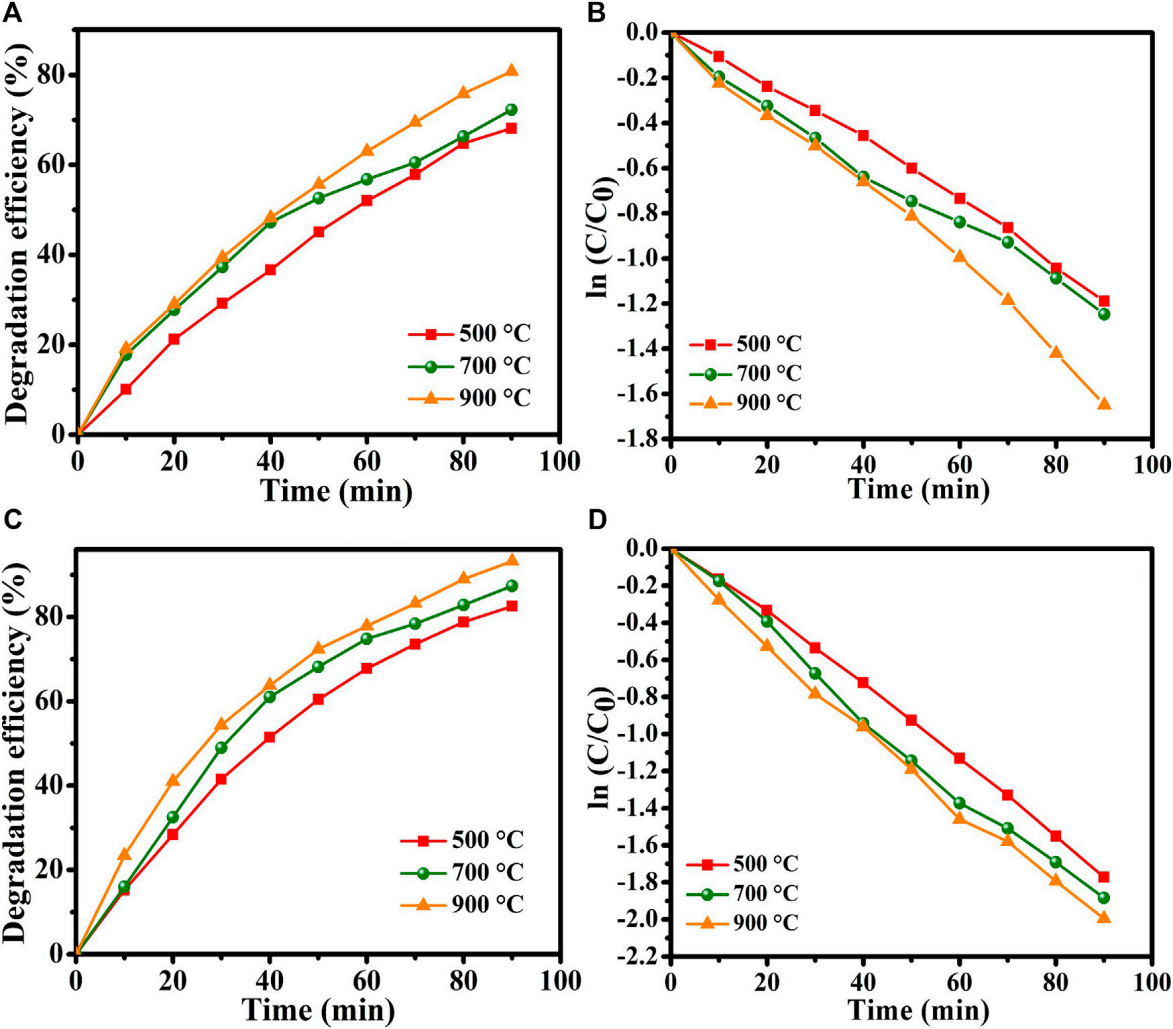
**(A)** Degradation efficiency of MO dye without H_2_O_2_, **(B)** photodegradation rate. **(C)** Degradation efficiency of MO dye with H_2_O_2_, **(D)** photodegradation rate.

Figure 8A displays the estimated degradation efficiency of MO dye in the absence of H_2_O_2_. The percentage degradation efficiency was calculated as,

$$Degradation\left(\%\right)=\frac{C_{0}-C}{C_{0}}\times 100\%$$

where C_0_ is the initial concentration of MO dye and C is the concentration of the dye following irradiation for time t. The graph in Figure 8A displays the degradation efficiencies of the samples annealed at 500°C, 700°C, and 900°C were found to 70.4%, 74.37%, and 81.3%, respectively. The sample annealed at 900°C shows the highest degradation efficiency revealing its superior photocatalytic activity. The increase in photodegradation efficiency can be attributed to a decrease in crystallite size, as agrees with the XRD results. Crystallite shrinkage increases the surface area-to-volume ratio of the catalyst, increasing the number of reactive sites and surface hydroxyl groups (Li et al., 2011). A large surface-area-to-volume ratio favors the reaction/interaction between the photocatalyst and the dye molecules, resulting in higher degradation efficiency. XRD results evidences that the samples annealed at 700°C and 900°C have similar phase structures and crystallinities. However, the sample annealed at 900°C exhibit a better photocatalytic performance whoci could be due to the existence of smaller crystallite size and more surface defects in materials. Smaller crystallite size corresponds to a larger total number of atoms with unsaturated coordinates on the surface, and these atoms have significantly improved photocatalytic activity. Additionally, the recombination of electrons and holes decreases as the crystallite size reduces, and thus the generated electrons and holes are transferred readily to the surface of the catalyst, favoring the process (Mageshwari et al., 2013).

To examine the surface area and porous structure of the prepared CuTa_2_O_6_ samples, BET surface analysis was carried out using nitrogen isothermal adsorption-desorption. Supplementary Figures S2A–C displays the isotherm curves of CuTa_2_O_6_ at different annealed temperatures along with the estimated pore-size distribution using the BJH method (Supplementary Figures S2D–F). The calculated specific surface area and BJH desorption Pore volume are presented in Supplementary Table S1. Generally, the surface area to volume ratio of nanomaterial plays a substantial role in influencing the material’s properties (Xia et al., 2012). When the particle size decreases, there is an enhancement in the surface area-to-volume ratio (Liu et al., 2015). Consequently, the sample annealed at 900°C shows a higher BET surface area (10.96 m^2^/g) than the other samples (9.07 m^2^/g for 500°C and 7.56 m^2^/g for 700°C), indicating a greater number of existing active sites. We, therefore, believe that the large specific surface area benefits better access and diffusion of liquid and gaseous reactants, which is beneficial for photocatalytic reactivity. The increased pore size distribution in the BJH desorption process is also likely to improve the photocatalytic performance of the materials (Supplementary Figures S2D–F).

The kinetics of the process importantly affect the rate at which pollution is removed. The kinetics of photocatalysis were determined from experimental data using the Langmuir—Hinshelwood model.

$$\ln\frac{C_{0}}{C}=K_{r}t$$

Here, K_r_ is the degradation rate constant and t is the degradation time. The degradation rate constant K_r_ is calculated from the slope of the kinetic plot of the natural logarithm of the concentration ratio ln (C/C_0_) versus the irradiation time t in minutes. Figure 8B plots this curve for MO dye in the absence of H_2_O_2_. The concentration ratio varies linearly with time suggest that the photodegradation of MO dye follows first-order kinetics. The degradation rate constants K_r_t for the samples that were annealed at 500°C, 700°C, and 900°C are calculated to be 0.01751 min^-1^, 0.01333 min^-1^, and 0.01107 min^-1^, respectively.

To enhance the photocatalytic activity of the prepared CuTa_2_O_6_, H_2_O_2_ (0.6 mL) was added as a green additive. Figure 8C shows the degradation efficiency of the samples with the addition of H_2_O_2_; they have higher degradation efficiencies than those achieved without H_2_O_2._ Therefore, the addition of H_2_O_2_ substantially enhanced photocatalytic activity. Then, Figure 8D displays the linear increase in the degradation rate. The degradation efficiencies of the samples that were annealed at 500°C, 700°C, and 900°C with added H_2_O_2_ are calculated to be 82.5%, 87.3%, and 94.7% with estimated rates constants of 0.01251 min^-1^, 0.01129 min^-1^ and 0.00866 min^-1^, respectively. Hence, the addition of H_2_O_2_ achieves the efficient photodegradation of MO in the presence of copper tantalum oxide.

The high-performance activity as a result of adding H_2_O_2_ can be explained as follows. H_2_O_2_ acts as a good electron acceptor, accelerating the reaction by increasing the formation of oxidizing radicals, which rapidly degrade the dye molecules. When the photons are incident upon the semiconductor, the photogenerated holes react with H_2_O or OH^−^ and produce hydroxyl radicals (^•^OH) while the superoxide (O_2_^−•^) and (^•^OH) radicals are generated by the electrons in the conduction band (e_CB_^−^). The produced O_2_^−•^ radicals further form hydroperoxy (OOH^•^) and ^•^OH radicals, which destroy the organic contaminants. The possible chemical reactions for the degradation of organic dyes are as follows.

Semiconductor+hγ → hVB++eCB−hVB++H2O →O−H+H+hVB++OH− →O•HeCB−+H2O2 →O•H+O−HeCB−+O2 → O2−•O2−•+H+ → HO2•H2O2+O2−• →O•H+OH−+O2H2O2+hγ → 2H2ODye+(•OH/O2−•/OOH•) → degradation

**FIGURE 9:**
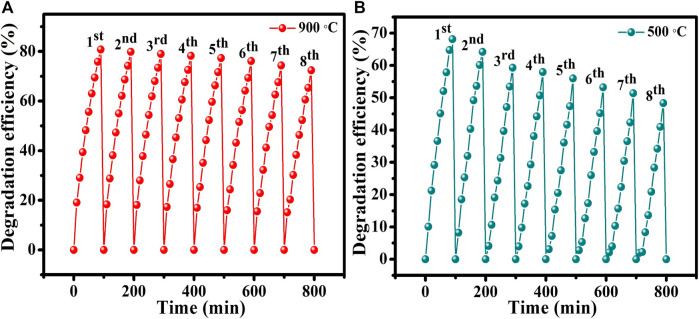
Recycling experiment of samples annealed at **(A)** 900°C and **(B)** 500°C.

To investigate the stability and reusability of the catalysts, an experiment on the recycling of the samples that were annealed at 500°C and 900°C was conducted. The catalyst sample annealed at 900°C underwent little change or deactivation, as shown in Figure 9A. The sample annealed at 500°C exhibits a considerable decrease in the efficiency of photodegradation, as shown in Figure 9B. Notably, the photodegradation efficiency experiences a rapid decline as early as the second and third runs. Therefore, it is believed that the cubic phase of CuTa_2_O_6_ is more stable for the photodegradation of MO dye than the orthorhombic phase (sample that was annealed at 500°C).

## 4 Conclusion

This work demonstrates the photocatalytic activity of the CuTa_2_O_6_ phase prepared at different annealing temperatures. The structure, morphology, optical properties, and elemental composition were studied using XRD, FESEM, UV-Vis DRS, and XPS. Structural studies confirmed the phase change from orthorhombic to cubic as the temperature of annealing increased. Both phases had the same morphology and exhibited significant visible light absorption. The cubic phase of CuTa_2_O_6_ supported a higher MO dye degradation efficiency than the orthorhombic phase. The local atomic and electronic structures around Cu and the oxidation state of Cu in the CuTa_2_O_6_ system were determined by X-ray absorption spectroscopy. The effect of the addition of H_2_O_2_ to the prepared samples was determined. The above results demonstrate that CuTa_2_O_6_ can be used as photocatalysts for environmental remediation. The prepared CuTa_2_O_6_ phase exhibits significant photocatalytic activity and could be a promising photocatalyst in water-splitting applications.

## Data Availability

The original contributions presented in the study are included in the article/Supplementary Material, further inquiries can be directed to the corresponding authors.

## References

[B1] Abdelrahman E. A. Hegazey R. Kotp Y. H. Alharbi A. (2019). Facile synthesis of Fe_2_O_3_ nanoparticles from Egyptian insecticide cans for efficient photocatalytic degradation of methylene blue and crystal violet dyes. Spectrochimica Acta Part A Mol. Biomol. Spectrosc. 222, 117195. 10.1016/j.saa.2019.117195 31176151

[B2] Ajmal A. Majeed I. Malik N. Idriss H. Nadeem M. A. (2014). Principles and mechanisms of photocatalytic dye degradation on TiO_2_ based photocatalysts: A comparative overview. RSC Adv. 4, 37003–37026. 10.1039/c4ra06658h

[B3] Bera P. Priolkar K. Sarode P. Hegde M. Emura S. Kumashiro R. (2002). Structural investigation of combustion synthesized Cu/CeO_2_ catalysts by EXAFS and other physical techniques: Formation of a Ce_1-x_Cu_x_O_2-δ_ solid solution. Chem. Mater. 14, 3591–3601. 10.1021/cm0201706

[B4] Chennakesavulu K. Ramajaneya Reddy G. (2015). Synthesis and characterization of carbon microtube/tantalum oxide composites and their photocatalytic activity under visible irradiation. RSC Adv. 5, 56391–56400. 10.1039/c5ra06812f

[B5] Djurišić A. B. Leung Y. H. Ching Ng A. M. (2014). Strategies for improving the efficiency of semiconductor metal oxide photocatalysis. Mater. Horizons. 1, 400. 10.1039/c4mh00031e

[B6] Dutta D. P. Ballal A. Fulekar M. K. Tyagi A. K. (2013). Multifunctionality of rare Earth doped nano ZnSb_2_O_6_, CdSb_2_O_6_ and BaSb_2_O_6_: Photocatalytic properties and white light emission. Dalton Trans. 42, 16887–16897. 10.1039/c3dt51966j 24091883

[B7] Gheytanzadeh M. Baghban A. Habibzadeh S. Jabbour K. Esmaeili A. Mohaddespour A. (2022). An insight into tetracycline photocatalytic degradation by MOFs using the artificial intelligence technique. Sci. Rep. 12, 6615–6711. 10.1038/s41598-022-10563-8 35459922 PMC9033875

[B8] Golubev A. Dinnebier R. E. Schulz A. Kremer R. K. Langbein H. Senyshyn A. (2017). Structural and magnetic properties of the trirutile-type 1D-Heisenberg anti-ferromagnet CuTa_2_O_6_ . Inorg. Chem. 56, 6318–6329. 10.1021/acs.inorgchem.7b00421 28481108

[B9] Gomes L. E. da Silva M. F. Goncalves R. V. Machado G. Alcantara G. B. Caires A. R. (2018). Synthesis and visible-light-driven photocatalytic activity of Ta^4+^ self-doped gray Ta_2_O_5_ nanoparticles. J. Phys. Chem. C 122 (11), 6014–6025. 10.1021/acs.jpcc.7b11822

[B10] Hodges B. C. Cates E. L. Kim J. (2018). Challenges and prospects of advanced oxidation water treatment processes using catalytic nanomaterials. Nat. Nanotechnol. 13, 642–650. 10.1038/s41565-018-0216-x 30082806

[B11] Kato M. Ishii T. Kajimoto K. Yoshimura K. Kosuge K. Nishi M. (2002). Magnetic properties of CuSb_2-x_Ta_x_O_6_ with tri-rutile structure. J. Phys. Chem. Solid. 63, 1129–1132. 10.1016/s0022-3697(02)00026-4

[B12] Kau L. S. Spira-Solomon D. J. Penner-Hahn J. E. Hodgson K. O. Solomon E. I. (1987). X-ray absorption edge determination of the oxidation state and coordination number of copper. Application to the type 3 site in Rhus vernicifera laccase and its reaction with oxygen. J. Am. Chem. Soc. 109, 6433–6442. 10.1021/ja00255a032

[B13] Khalid N. R. Ahmed E. Hong Z. Ahmad M. Zhang Y. Khalid S. (2013). Cu-doped TiO_2_ nanoparticles/graphene composites for efficient visible-light photocatalysis. Ceram. Int. 39, 7107–7113. 10.1016/j.ceramint.2013.02.051

[B14] Khemthong P. Photai P. Grisdanurak N. (2013). Structural properties of CuO/TiO_2_ nanorod in relation to their catalytic activity for simultaneous hydrogen production under solar light. Int. J. hydrogen energy 38, 15992–16001. 10.1016/j.ijhydene.2013.10.065

[B15] Krabbes I. Langbein H. (1996). Herstellung von CuTa_2_O_6_ von der Trirutil-zur Perowskit- Struktur. Zeit. Naturf. 51, 1605–1610. 10.1515/znb-1996-1113

[B16] Li J. Sun F. Gu K. Wu T. Zhai W. Li W. (2011). Applied Catalysis A: General Preparation of spindly CuO micro-particles for photodegradation of dye pollutants under a halogen tungsten lamp. Appl. Catal. A, Gen. 406, 51–58. 10.1016/j.apcata.2011.08.007

[B17] Li M. Zhang H. Zhao Z. Wang P. Li Y. Zhan S. (2023). Inorganic ultrathin 2D photocatalysts: Modulation strategies and environmental/energy applications. Acc. Mater. Res. 4 (1), 4–15. 10.1021/accountsmr.2c00172

[B18] Liang P. Zhang C. Sun H. Liu S. Tade M. Wang S. (2017). Solar photocatalytic water oxidation and purification on ZIF-8-derived C–N–ZnO composites. Energy & Fuels 31, 2138–2143. 10.1021/acs.energyfuels.6b02057

[B19] Lim H. Thomas K. E. Hedman B. Hodgson K. O. Ghosh A. Solomon E. I. (2019). X-Ray absorption spectroscopy as a probe of ligand noninnocence in metallocorroles: The case of copper corroles. Inorg. Chem. 58, 6722–6730. 10.1021/acs.inorgchem.9b00128 31046257 PMC6644708

[B20] Liu H. Gou X. Wang Y. Du X. Quan C. Qi T. (2015). Cauliflower-like Co_3_O_4_/three-dimensional graphene composite for high performance supercapacitor applications. J. Nanomater. 2015, 1–9. 10.1155/2015/874245

[B21] Louis J. Padmanabhan N. T. Jayaraj M. K. John H. (2022). Crystal lattice engineering in a screw-dislocated ZnO nanocone photocatalyst by carbon doping. Mater. Adv. 3 (3), 4322–4333. 10.1039/d2ma00098a

[B22] Mageshwari K. Sathyamoorthy R. Patil P. S. (2013). Template-free synthesis of MgO nanoparticles for effective photocatalytic applications. Powder Technol. 249, 456–462. 10.1016/j.powtec.2013.09.016

[B23] Majhi D. Mishra A. K. Das K. Bariki R. Mishra B. G. (2021). Plasmonic Ag nanoparticle decorated Bi2O3/CuBi2O4 photocatalyst for expeditious degradation of 17α-ethinylestradiol and Cr(VI) reduction: Insight into electron transfer mechanism and enhanced photocatalytic activity. Chem. Eng. J. 413, 127506. 10.1016/j.cej.2020.127506

[B24] Muthu Gnana Theresa Nathana D. Jacob Melvin Boby S. Basu P. Mahesh R. Harish S. Joseph S. (2018). One-pot hydrothermal preparation of Cu_2_O-CuO/rGO nanocomposites with enhanced electrochemical performance for supercapacitor applications. Appl. Surf. Sci. 449, 474–484. 10.1016/j.apsusc.2017.12.199

[B25] Nasir M. Patra N. Ahmed M. A. Shukla D. K. Kumar S. Bhattacharya D. (2017). Role of compensating Li/Fe incorporation in Cu_0.945_Fe_0.055-x_Li_x_O: Structural, vibrational and magnetic properties. RSC Adv. 7, 31970–31979. 10.1039/c7ra03960c

[B26] Nguyen H. Petitbon F. Fabry P. (1996). Investigations on the mixed conductivity of copper tantalate. Solid State Ionics 92, 183–192. 10.1016/s0167-2738(96)00492-4

[B27] Noto L. L. Chitambo M. L. Ntwaeaborwa O. M. Swart H. C. (2013). Photoluminescence and thermoluminescence properties of Pr^3+^ doped ZnTa_2_O_6_ phosphor. Powder Technol. 247, 147–150. 10.1016/j.powtec.2013.07.012

[B28] Oyewo O. A. Ramaila S. Mavuru L. Onwudiwe D. C. (2022). Enhanced photocatalytic degradation of methyl orange using Sn-ZnO/GO nanocomposite. J. Photochem. Photobiol. 11, 100131. 10.1016/j.jpap.2022.100131

[B29] Ramchiary A. (2020). “Metal-oxide semiconductor photocatalysts for the degradation of organic contaminants,” in Handbook of smart photocatalytic materials (Elsevier), 23–38.

[B30] Rose A. Hofmann A. Voepel P. Milow B. Marschall R. (2022). Photocatalytic activity and electron storage capability of TiO2 aerogels with an adjustable surface area. ACS Appl. Energy Mater. 5, 14966–14978. 10.1021/acsaem.2c02517

[B31] Sahoo D. Shakya J. Ali N. Yoo W. J. Kaviraj B. (2022). Edge rich ultrathin layered MoS2 nanostructures for superior visible light photocatalytic activity. Langmuir 38 (4), 1578–1588. 10.1021/acs.langmuir.1c03013 35072482

[B32] Sibhatu A. K. Weldegebrieal G. K. Sagadevan S. Tran N. N. Hessel V. (2022). Photocatalytic activity of CuO nanoparticles for organic and inorganic pollutants removal in wastewater remediation. Chemosphere 300, 134623. 10.1016/j.chemosphere.2022.134623 35439489

[B33] Sullivan I. Zoellner B. Maggard P. A. (2016). Copper(I)-Based p-type oxides for photoelectrochemical and photovoltaic solar energy conversion. Chem. Mater. 28 (17), 5999–6016. 10.1021/acs.chemmater.6b00926

[B34] Suzuki T. M. Saeki S. Sekizawa K. Kitazumi K. Takahashi N. Morikawa T. (2017). Photoelectrochemical hydrogen production by water splitting over dual-functionally modified oxide: p-Type N-doped Ta_2_O_5_ photocathode active under visible light irradiation. Appl. Catal. B Environ. 202, 597–604. 10.1016/j.apcatb.2016.09.066

[B35] Svintsitskiy D. A. Chupakhin A. P. Slavinskaya E. M. Stonkus O. A. Stadnichenko A. I. Koscheev S. V. (2013). Study of cupric oxide nanopowders as efficient catalysts for low-temperature CO oxidation. J. Mol. Catal. A Chem. 368, 95–106. 10.1016/j.molcata.2012.11.015

[B36] Tao C. Xu L. Guan J. (2013). Well-dispersed mesoporous Ta_2_O_5_ submicrospheres: Enhanced photocatalytic activity by tuning heating rate at calcination. Chem. Eng. J. 229, 371–377. 10.1016/j.cej.2013.06.012

[B37] Vincent H. Bochu B. Aubert J. J. Joubert J. C. Marezio M. (1978). Structure cristalline de CuTa_2_O_6_ . J. Solid State Chem. 24, 245–253. 10.1016/0022-4596(78)90016-6

[B38] Wang Z. Wang J. Hou J. Huang K. Jiao S. Zhu H. (2012). Facile synthesis of efficient photocatalytic tantalum nitride nanoparticles. Mater. Res. Bull. 47, 3605–3611. 10.1016/j.materresbull.2012.06.054

[B39] Xia H. Wang Y. Lin J. Lu L. (2012). Hydrothermal synthesis of MnO_2_/CNT nanocomposite with a CNT core/porous MnO_2_ sheath hierarchy architecture for supercapacitors. Nanoscale Res. Lett. 7, 33. 10.1186/1556-276x-7-33 24576342 PMC3292840

[B40] Xu L. Li C. Shi W. Guan J. Sun Z. (2012b). Visible light-response NaTa_1-x_Cu_x_O_3_ photocatalysts for hydrogen production from methanol aqueous solution. J. Mol. Catal. A, Chem. 360, 42–47. 10.1016/j.molcata.2012.04.006

[B41] Xu L. Steinmiller E. M. Skrabalak S. E. (2012a). Achieving synergy with A potential photocatalytic Z-scheme: Synthesis and evaluation of nitrogen-doped TiO_2_/SnO_2_ composites. J. Phys. Chem. C 116, 871–877. 10.1021/jp208981h

[B42] Yang X. Zhang S. Li P. Gao S. Cao R. (2020). Visible-light-driven photocatalytic selective organic oxidation reactions. Mater. Chem. A 8, 20897–20924. 10.1039/d0ta05485b

[B43] Zhang L. S. Wang W. Z. Yang J. Chen Z. G. Zhang W. Q. Zhou L. (2006). Sonochemical synthesis of nanocrystallite Bi_2_O_3_as A visible-light-driven photocatalyst. Appl. Catal. A Gen. 308, 105–110. 10.1016/j.apcata.2006.04.016

[B44] Zhang P. Zhang J. Gong J. (2014). Tantalum-based semiconductors for solar water splitting. Chem. Soc. Rev. 43, 4395–4422. 10.1039/C3CS60438A 24668282

[B45] Zhou L. Wang W. Z. Xu H. L. Sun S. M. Shang M. (2009). Bi_2_O_3_ hierarchical nanostructures: Controllable synthesis, growth mechanism, and their application in photocatalysis. Chem.Eur. J. 15, 1776–1782. 10.1002/chem.200801234 19115297

