# Supplementary material for: Synthesis and characterization of visible-light-driven novel CuTa2O6 as a promising practical photocatalyst
Source: Front Chem. 2023 Jun 22;11:1197961. doi: 10.3389/fchem.2023.1197961 (PMC10323133; doi:10.3389/fchem.2023.1197961)
Supplement: Supplementary file 1 [file Presentation1.pdf]

Supplementary material

Synthesis and characterization of visible-light-driven novel $CuTa_2O_6$ as a promising practical photocatalyst

Krishnaprasanth Alageshwaramoorthy[1], Pandian Mannu[1], Seetha Mahalingam[1*], Ta Thi Thuy Nga[2], Han-Wei Chang[3,4], Yoshitake Masuda[5], Chung-Li Dong*[2]

[1] Department of Physics, Kongunadu Arts and Science College, Coimbatore 641 029, India

[2] Research Center for X-ray Science & Department of Physics, Tamkang University, Tamsui 25137, Taiwan

[3] Department of Chemical Engineering, National United University, Miaoli 360302, Taiwan

[4] Pesticide Analysis Center, National United University, Miaoli 360302, Taiwan

[5] National Institute of Advanced Industrial Science and Technology (AIST), Nagoya 463 8560, Japan

Corresponding author:

M. Seetha: seetha.phy@gmail.com

C. L. Dong: cldong@mail.tku.edu.tw

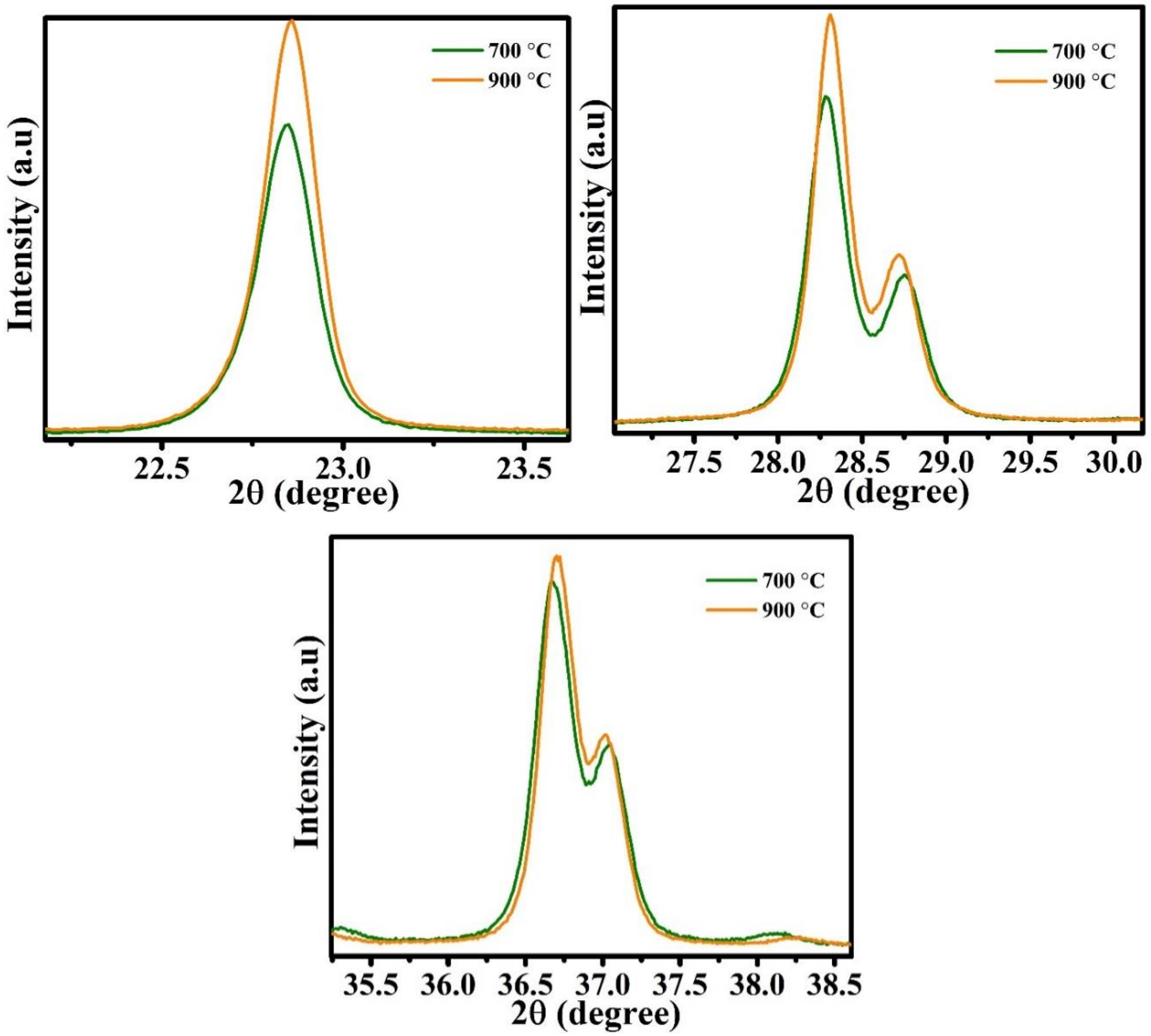

Figure S1. The XRD peak positions of $CuTa_2O_6$ at different angles.

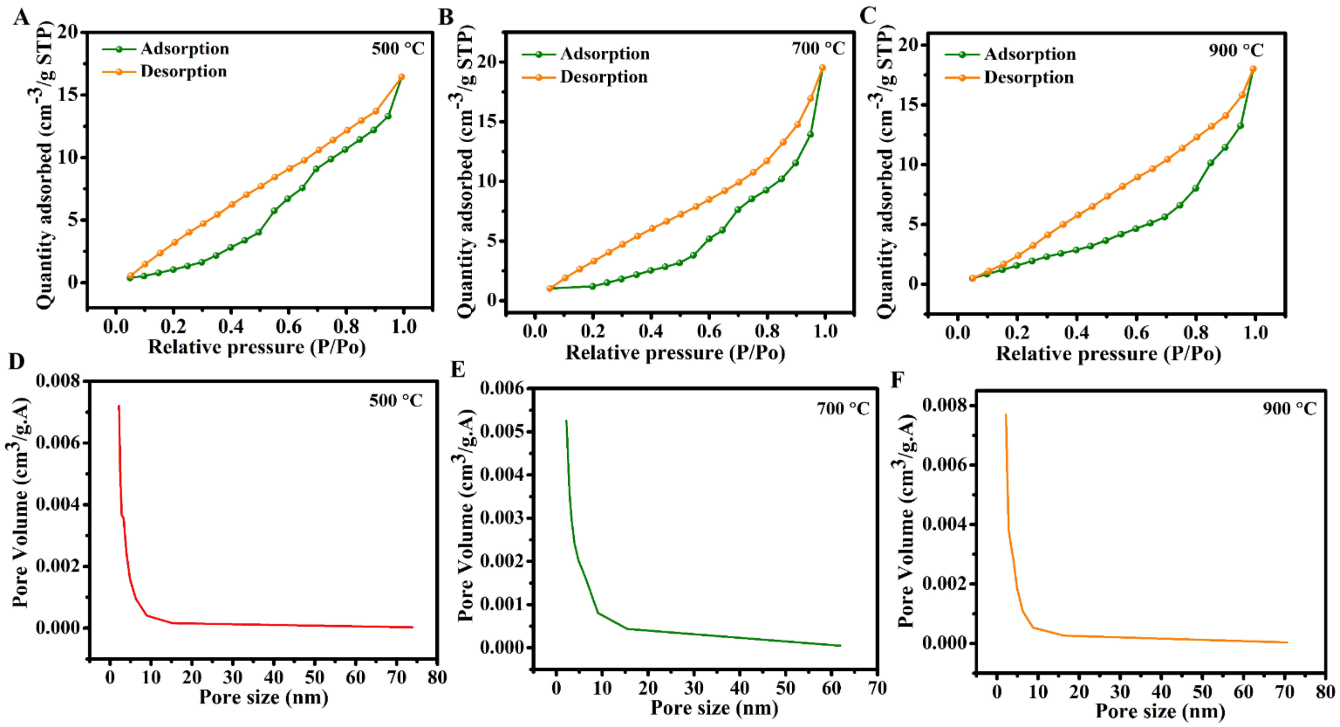

Figure S2. (A, B, & C) BET surface area measured from N_2 adsorption-desorption isotherms and (D, E, & F) pore volume distribution from desorption isotherms of $CuTa_2O_6$ photocatalysts.

Table S1. The calculated surface area and pore size of $CuTa_2O_6$ samples

**Sample**	**BET Surface Area (m^2/g)**	**BJH desorption Pore Volume (cc/g)**
**500º C**	7.565	0.0241301
**700º C**	9.077	0.0277883
**900º C**	10.962	0.0284469
